# Auditory brainstem response prior to MRI compared to standalone MRI in the detection of vestibular schwannoma: A modelling study

**DOI:** 10.1111/coa.13894

**Published:** 2021-11-24

**Authors:** Stan R. W. Wijn, Mayke A. Hentschel, Andy J. Beynon, Henricus P. M. Kunst, Maroeska M. Rovers

**Affiliations:** ^1^ Department of Operating Rooms Radboud Institute of Health Sciences, Radboud University Medical Centre Nijmegen The Netherlands; ^2^ Department of Otolaryngology Radboud Institute of Health Sciences, Radboud University Medical Centre Nijmegen The Netherlands; ^3^ Academic Alliance Skull Base Pathology, Radboud University Medical Centre & Maastricht University Medical Centre Nijmegen & Maastricht The Netherlands; ^4^ Department of Otorhinolaryngology Maastricht University Medical Centre Maastricht The Netherlands; ^5^ Department of Health Evidence Radboud Institute of Health Sciences, Radboud University Medical Centre Nijmegen The Netherlands

**Keywords:** auditory brainstem response, cost‐effectiveness analysis, diagnostic, magnetic resonance imaging, vestibular schwannoma

## Abstract

**Objectives:**

To determine the cost‐effectiveness of auditory brainstem response prior to MRI (ABR‐MRI) compared to standalone MRI to diagnose vestibular schwannoma.

**Design:**

A state transition model was developed to simulate costs and effects (quality‐adjusted life years [QALY]) for both diagnostic strategies for patients suspected of a vestibular schwannoma. Model input was derived from literature, hospital databases and expert opinions. Scenario and sensitivity analyses addressed model uncertainty.

**Results:**

Over a lifetime horizon, ABR‐MRI resulted in a limited cost‐saving of €68 or €98 per patient (dependent on MRI sequence) and a health loss of 0.005 QALYs over standalone MRI. ABR‐MRI, however, did miss patients with other important pathology (2% of the population) that would have been detected when using standalone MRI. In total, €14 203 or €19 550 could be saved per lost QALY if ABR‐MRI was used instead of standalone MRI. The results were sensitive to the detection rate of vestibular schwannoma and health‐related quality of life of missed patients.

**Conclusion:**

The cost‐saving with ABR‐MRI does not seem to outweigh the number of missed patients with VS and other important pathologies that would have been detected when using standalone MRI.

**Key points**

- A state transition model was developed to simulate costs and effects for ABR prior to MRI and standalone MRI for patients suspected of a vestibular schwannoma.
- The cost‐saving with ABR‐MRI does not seem to outweigh the missed patients with vestibular schwannoma and other important pathologies.
- ABR‐MRI missed patients with other important pathology that would have been detected when using standalone MRI.
- The cost‐saving with ABR‐MRI was only worthwhile in scenarios with no negative consequences for missed patients.
- Sensitivity analyses showed that the cost‐effectiveness of ABR‐MRI is sensitive to the MRI sequence, the probability that a missed patient is detected during follow‐up and the quality of life of a missed patient.

## 1 INTRODUCTION

Vestibular schwannoma (VS) is a benign intracranial tumour growing on the myelin‐forming cells of the hearing and balance nerves of the inner ear (8th cranial nerve).^1^ The overall incidence is about two per 100.000 person‐years but is slowly increasing due to the general use of diagnostic imaging techniques such as magnetic resonance imaging (MRI).^1^ Patients with symptoms of VS are usually screened with MRI (contrast‐enhanced T1 weighted [T1W] and/or T2 weighted [T2W]) of the brain and cerebellopontine angle.^2^, ^3^ Consequently, VS is confirmed in approximately 3% of patients with symptoms.^4^ Using MRI, other pathologies aside from VS‐like cysts, aneurysms, infarctions and malignancies can be diagnosed. However, approximately 84% of the scans do not reveal any pathology.^4^ Due to this low incidence of VS, this diagnostic pathway is costly. To reduce the costs, hospitals frequently use auditory brainstem response (ABR) as a preliminary diagnostic method to select high‐risk VS patients prior to MRI.^5^, ^6^, ^7^ In a survey performed in European ear‐nose‐throat physicians, nearly 50% indicated that they still use ABR as a preliminary diagnostic method prior to MRI for VS (ABR‐MRI).^8^ ABR, however, has a lower sensitivity and specificity compared to MRI, especially for the detection of small VSs (<1 cm) and will miss patients with VS and other pathology.

There seems to be a paradigm shift from active treatment to prolonged periods of W &S even when (small) tumour growth is present. This is mainly based on the improved understanding of limited VS growth and small differences in health‐related quality of life (HRQoL) of the different treatment strategies.^9^ Therefore, the impact of a missed case of VS with ABR is arguably reduced, increasing the likelihood that ABR‐MRI is cost‐effective compared to standalone MRI. Several studies have been performed to measure the (cost‐) effectiveness of ABR as a diagnostic tool. However, most studies were outdated,^7^, ^10^, ^11^ did not include an effect measure,^6^, ^12^ or did not include the consequence of a missed case when ABR would be used as a screening tool prior to MRI. Therefore, we aimed to evaluate the cost‐effectiveness of ABR screening prior to MRI compared to standalone MRI for the detection of VS.

## 2 METHODS

### 2.1 Ethical considerations

This modelling study was based on the published literature and did not involve human subjects, and therefore, ethical approval or informed consent was not required.

### 2.2 Decision‐analytic model

The differences between the ABR‐MRI strategy and the current standalone MRI strategy were mapped using a decision‐analytic model. A decision‐analytic model offers a framework to synthesise the available evidence about probabilities, effects and/or costs and to combine it with a degree of uncertainty and was performed from a health care perspective. This study was designed according to the Consolidated Health Economic Evaluation Reporting Standards (CHEERS) Statement^13^ and built using Microsoft Excel 2016 (Redmond, Washington, United States of America).

### 2.3 Overview of the model

**FIGURE 1:**
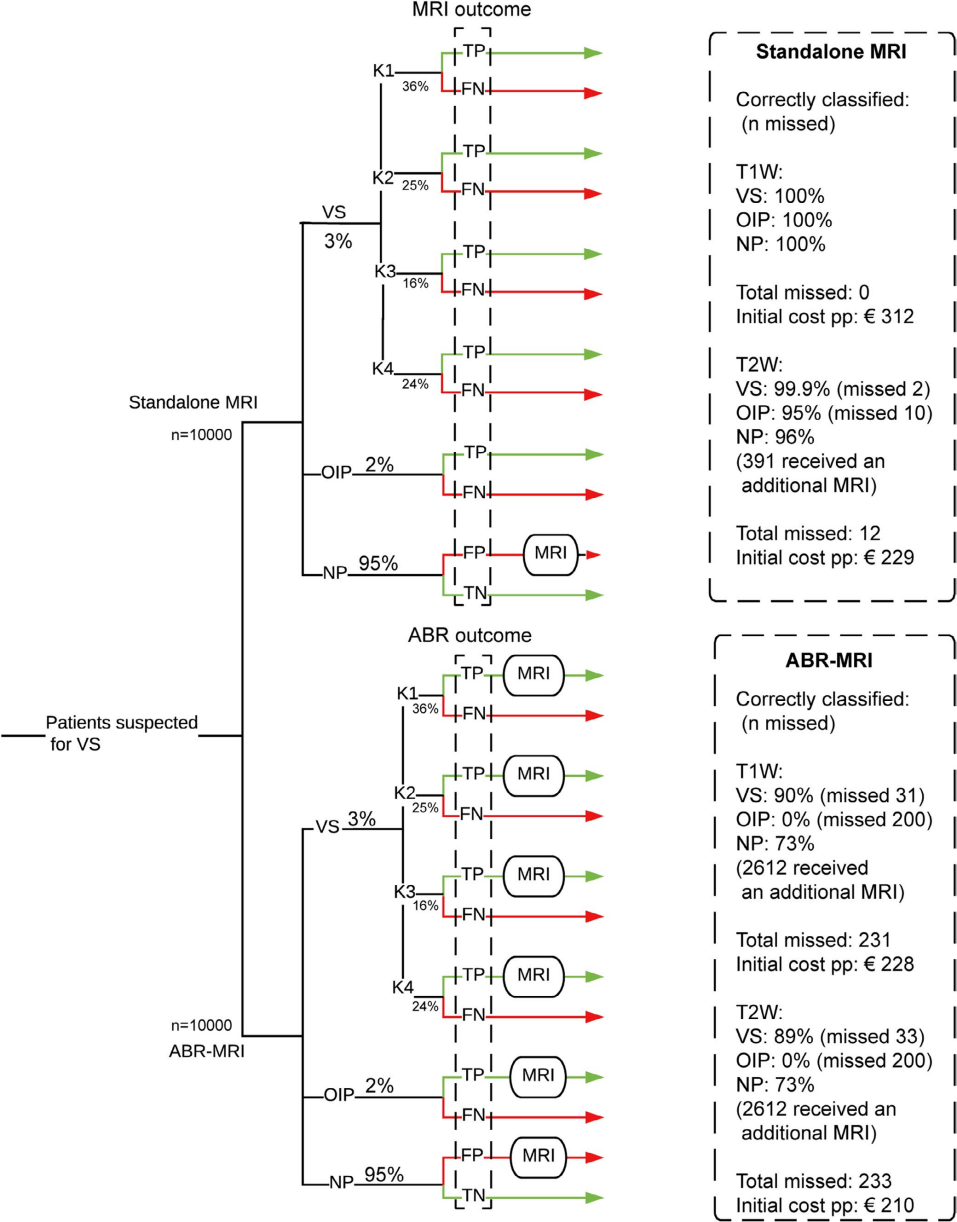
Decision‐tree comparing ABR‐MRI with standalone MRI as a diagnostic tool to detect VS. The upper branch is the standalone MRI strategy and the bottom branch is the ABR‐MRI strategy. ABR, auditory brainstem response; FN, false negative; FP, false positive; K1‐K4, Koos stages;^16^ MRI, magnetic resonance imaging; NP, no pathology; OIP, other important pathology; TN; true negative; TP, true positive; VS, vestibular schwannoma

We developed the model to determine the costs and effects of both strategies to diagnose patients suspected of VS based on an existing model for VS.^14^ Patients were defined as suspected of VS if they reported asymmetrical audiovestibular symptoms including asymmetrical sensorineural hearing loss, tinnitus or vertigo. A hypothetical cohort of 10 000 patients with symptoms of VS went through the model for each strategy (Figure 1 and Appendix S1). The cohort started at age 50, the mean age of the population.^15^ The model started by dividing the patient population according to the disease group: patients with VS, patients with other important pathology (OIP) that require treatment (like aneurysms and intracranial malignancies), and patients (NP) that do not require treatment (like atrophy of the brain or infarctions).^4^ Previous studies have shown that T2W MRI sequence is cheaper compared to T1W sequence but will miss some patients due to lower sensitivity.^3^ ABR’s sensitivity mainly depends on the size of the tumour.^5^, ^6^, ^10^ Therefore, both MRI sequences were compared to ABR, and all VS patients were classified according to tumour size using the Koos classification system.^16^

**FIGURE 2:**
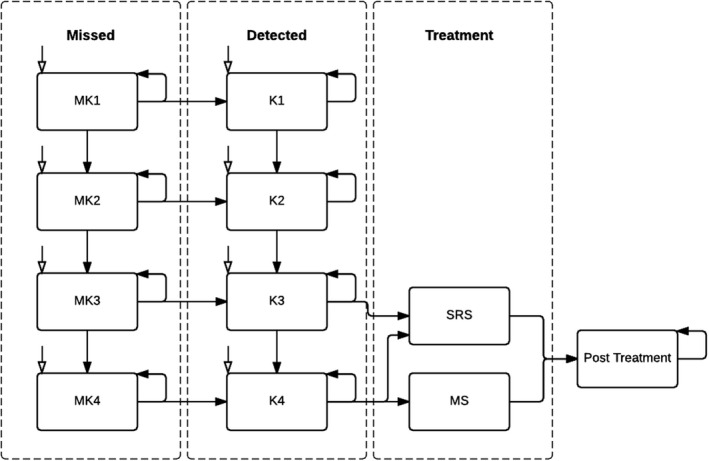
Markov chain simulation comparing long‐term costs and effects of ABR with MRI as a diagnostic tool to detect VS with 13 health states. White arrows indicate model entry. From every state, patients can transition to the death state. Death state, other important pathology, and no pathology group were not depicted. MK1‐4, missed Koos 1–4; K1‐4, detected Koos 1–4; SRS, stereotactic radiosurgery; MS, microsurgery

The long‐term consequences were simulated using a Markov chain simulation. Using cycles to simulate time, patients transitioned between so‐called health states. Every cycle of the model simulated 1 year and ended after a lifetime, at which all patients transferred to the death state. The Markov chain simulation (Figure 2) consisted of 13 health states, for every detected and undetected Koos stage, treatment states and post‐treatment state, including an absorbing state marking death.

### 2.4 Transition probabilities

All probabilities of the decision model were based on the literature or, if unavailable, on consensus expert opinion (Table 1). Parameters from the literature were collected using a detailed search of Pubmed and EMBASE. The consensus expert opinion was constructed by two clinical experts. The patient population was divided according to the disease group using incidences.^4^ The tumour size probability was 0.356, 0.247, 0.161 and 0.236 for Koos 1 to 4, respectively.^17^ The probability of tumour growth and transition to the next Koos stage was the largest in the first year after diagnosis (10%) and gradually decreased every year (5%, 2.5%, 1.5%).^1^ After four years the annual probability of transitioning to the next Koos stage was assumed to be 0.18%, until 30 years after initial diagnostics, when growth was assumed not to occur.^1^ When a patient with VS was missed, we assumed that they had a yearly 10% chance to be detected based on the assumption that the majority of all missed patients with VS will receive additional diagnostics in the upcoming years (after 10 years, 65% is detected).

**TABLE 1:** Transition probabilities used in the cost‐effectiveness model, including the 95% confidence interval

Parameter	Point estimate	95% Confidence interval	Source
Probabilities			
Vestibular schwannoma (VS)	0.03	(0.02–0.04)	Dawes [2000]^4^
Other important pathology (OIP)	0.02	(0.01–0.03)	Dawes [2000] ^4^
No pathology (NP)	0.95	Dependent on other parameters	Dawes [2000]^4^
Sensitivity MRI T1W + Gd	1.00	(0.98–1.00)	Ahmad [2014]/Fortnum [2009]^2^, ^3^
Specificity MRI T1W + Gd	1.00	(0.98–1.00)	Ahmad [2014]/Fortnum [2009]^2^, ^3^
Sensitivity MRI T2W	0.98	(0.94–0.99)	Fortnum [2009]^3^
Specificity MRI T2W	0.96	(0.94–0.96)	Fortnum [2009]^3^
Sensitivity other pathology MRI	0.95	(0.80–1.00)	Expert opinion
Specificity ABR	0.82	(0.805–0.836)	Koors [2012]^5^
Sensitivity ABR tumours <1cm	0.85	(0.806–0.901)	Fortnum [2009]^3^
Sensitivity ABR tumours >1cm	0.95	(0.931–0.982)	Fortnum [2009]^3^
Sensitivity other pathology ABR	0.00	(0.0–0.3; uniform distribution)	Expert opinion

		Assumptions/95% Confidence interval	
Koos 1	0.356	(0.339–0.374)	Hentschel [2020]^17^
Koos 2	0.247	(0.235–0.259)	Hentschel [2020]^17^
Koos 3	0.161	(0.153–0.169)	Hentschel [2020]^17^
Koos 4	0.236	(0.224–0.247)	Hentschel [2020]^17^
Growth to other Koos stage	Cycle‐dependent	In the first 4 years, the probabilities were 0.10, 0.05, 0.025, and 0.015. We linearly extrapolated this pattern up to 30 years, with an annual probability of 0.0018. After 30 years, growth was assumed not to occur.	Stangerup [2006]^1^
Chance of detecting a missed VS	10%/year	We assumed that a missed case had a 10% chance each year to be detected. (0.00–1.00)	
Death after MS	0.01		Huang [2017]^18^
Microsurgery for Koos 4 tumours	0.9		Expert opinion
Death	Cycle‐dependent		Central Agency for Statistics (CBS)^19^

All parameters originate from literature sources or expert opinions.

Abbreviations: ABR, auditory brain response; Gd, gadolinium; MRI, magnetic resonance imaging; MS, microsurgery; NP, no pathology; OIP, other important pathology; T1W, T1‐weighted MRI sequence; T2W, T2‐weighted MRI sequence; VS, vestibular schwannoma.

### 2.5 Costs

All costs were drawn from cost‐effectiveness guidelines, hospital tariffs or by reaching consensus with experts (Table 2).^20^, ^21^ To calculate the cost of an MRI T1W sequence, the extra cost of gadolinium was added to the cost of a normal MRI brain, including a margin to cover extra setup and sequence times, estimated at €94. Patients with detected VS in the W&S strategy were annually monitored with MRI until death. All costs were indexed to 2020.

**TABLE 2:** Cost parameters used in the cost‐effectiveness model

Parameter	Estimated value	Assumption	Source
Costs			
MRI brain (T2W)	€218		Dutch guideline for cost‐effectiveness studies^21^
MRI brain with contrast (T1W)	€312*		Dutch guideline for cost‐effectiveness studies^21^ + €94*
MRI retest (T2W) + visit	€318	The retests include the cost for the MRI and an additional visit to the ENT department.	Expert opinion
MRI retest (T1W) + visit	€412		Expert opinion
ABR	€154		Dutch guideline for cost‐effectiveness studies^21^
Microsurgery	€14 689		Hospital Tariff^20^
Stereotactic radiosurgery	€9577		Hospital Tariff^20^
Cost after treatment	€191		Expert opinion

Abbreviations: ABR, auditory brain response; ENT, ear‐nose‐throat; MRI, magnetic resonance imaging; T1W, T1‐weighted MRI sequence; T2W, T2‐weighted MRI sequence.

* The costs of a T1W MRI sequence with gadolinium were not listed and the authors were not able to identify the exact costs. However, assuming that a standard MRI is €218 and the estimated costs for a vial of gadolinium are €50, including a margin to cover extra setup and sequence times, the additional costs of the T1W sequence with gadolinium were set at €94.

### 2.6 Effects

To measure the effectiveness of both strategies, the number of missed cases were calculated and the differences in HRQoL between the patient groups using quality‐adjusted life years (QALYs). The QALY is a preferred health outcome including both quality and quantity of life. To calculate a QALY, the HRQoL is transformed into a utility score. A utility score is a numeric value ranging from death (0) to perfect health (1). Utility scores of the different health states were derived from literature and expert opinion (Table 3). HRQoL scores derived from the 36‐Item Short Form Survey (SF‐36) were converted to utilities.^22^ The utility scores of missed VS patients were assumed to be equal to the detected patients, while the disutility of missed patients with OIP were assumed to be 0.01.

**TABLE 3:** Utility parameters used in the cost‐effectiveness model. Utilities range from 0 to 1, presenting a scale from death to full health

Parameter	Estimated utility value	Confidence interval/Assumption	Source
Utility			
Koos 1	0.83	(0.79–0.87)	Hentschel [2020]^23^
Koos 2	0.82	(0.76–0.88)	Hentschel [2020]^23^
Koos 3	0.77	(0.69–0.85)	Hentschel [2020]^23^
Koos 4	0.76	(0.65–0.86)	Hentschel [2020]^23^
Missed patient (base case)	+0.00	Equal to detected patients	Assumption
Missed patient (assumption 2)	−0.05	Lower compared to detected patients	Assumption
Missed patient (assumption 3)	+0.05	Higher compared to detected patients	Assumption
First‐year after SRS	0.75		Gait [2014]^24^
First‐year after MS	0.70		Godefroy[2007]^25^
Post‐treatment	0.80	(0.60–0.90)	Cheng [2009]/Godefroy[2007]^11^, ^25^
OIP/NP	0.83	Patients with VS‐like symptoms are assumed to have a utility value equal to missed Koos 1 patients	Expert opinion
Missed OIP	−0.01	Missed patients with other important pathology were assumed to have a disutility of 0.01	Assumption

Abbreviations: MS, microsurgery; NP, no pathology; OIP, other important pathology; SRS, stereotactic radiosurgery.

### 2.7 Assumptions

Every cost‐effectiveness model is restricted by assumptions in order to be functional and to improve comprehensibility. We assumed that all patients were eligible for both ABR and MRI and accepted the prescribed diagnostic test. In the case of a positive ABR result, we assumed that MRI would subsequently identify it as false‐positive, independently of the sensitivity and specificity of MRI. There was no loss of patients during the diagnostic process or during the follow‐up of patients with VS because we did not encounter evidence of systematic refusal to perform diagnostic tests in this patient category.

### 2.8 Analysis

We compared the number of missed cases, expected costs and effects over a lifetime for the ABR‐MRI strategy and the standalone MRI strategy for both MRI sequences. According to guidelines, all costs and effects were discounted with a 4% rate for costs and 1.5% rate for effects.^21^ To determine the cost‐effectiveness of the two diagnostic strategies, the incremental cost‐effectiveness ratio (ICER) was calculated by dividing the incremental cost by the incremental effect.

### 2.9 Scenario and sensitivity analyses

In the base case analysis, we assumed that the HRQoL of missed VS patients was equal to detected patients. In the scenario analyses, these utilities were varied to include a scenario in which we assumed that the lack of a diagnosis reduces the utility score (−0.05) or improves the utility score (+0.05) compared to detected patients.

To explore the influence of uncertainty on the parameter estimates, sensitivity analyses were performed. For the univariate sensitivity analyses, we varied (1) the probability of detecting a missed VS patient after one cycle and (2) the disutility score of a patient with OIP that receives a false‐negative diagnosis, to determine the impact of a missed case in need of treatment. We varied the probability of detecting a missed VS patient between 0% and 100% using five intervals, and the disutility score of a patient with OIP was varied from 0.00 to 0.05. The probabilistic sensitivity analysis consisted of 10 000 (Monte Carlo) simulations to reflect the sampling uncertainty, drawn from beta distributions (Tables 1 and 3).

## 3 RESULTS

### 3.1 **Base** model analysis

At both the T1W and T2W MRI sequence, ABR‐MRI saved costs but missed more patients with VS or OIP compared to standalone MRI. The initial costs in the T1W MRI sequence were €228 per patient and €312 (ABR‐MRI and standalone MRI, respectively), but ABR‐MRI missed 31 of the 300 patients with VS (10%; Koos1: 15, Koos2: 11, Koos3: 2, Koos4: 3) and all patients with OIP (200 patients, 2% of the total population), compared to no missed cases in the standalone MRI strategy. The initial costs in the T2W MRI sequence were €210 and €229 (ABR‐MRI and standalone MRI, respectively). ABR‐MRI missed 33 of the 300 patients with VS (11%; Koos1: 17, Koos2: 11, Koos3: 2, Koos4: 3) and all patients with OIP (200 patients, 2% of total population), while standalone MRI missed 2 of the 300 patients with VS (0.6%; both Koos1) and 10 of the 200 patients with OIP (5%).

Calculating costs and QALYs over time, ABR‐MRI showed a health loss of 0.005 QALYs over standalone MRI in both scenarios. In the T1W scenario, ABR‐MRI cost €636 per patient compared to €734 for standalone MRI, resulting in an ICER of €19 550 saved per QALY lost. In the T2W scenario, ABR‐MRI cost €516 compared to €583 for standalone MRI, resulting in an ICER of €14 203 saved per QALY lost.

### 3.2 Scenario analysis

Assuming that all missed patients had a lower HRQoL compared to detected patients resulted in an ICER of €3198 and €2322 per QALY lost for T1W and T2W scenario, respectively, indicating that standalone MRI is cost‐effective over ABR‐MRI. Assuming that all missed patients had a higher HRQoL compared to detected patients resulted in the domination of ABR‐MRI over standalone MRI.

### 3.3 Univariate sensitivity analysis

The probability of detecting a missed VS was assumed to be 10% per year, but varying this probability did not result in substantial cost savings. The highest ICER detected was €24 078 saved per QALY lost. When the disutility of a missed case of OIP was set to 0, the ICER increased to €860 682 and €594 764 for the T1W and T2W scenario, respectively, favouring ABR‐MRI. When the disutility of a missed case of OIP was doubled (−0.02, compared to the base case) the ICER dropped to €9887 and €7188 for the T1W and T2W scenario, respectively, favouring standalone MRI.

### 3.4 Probabilistic sensitivity analysis

**FIGURE 3:**
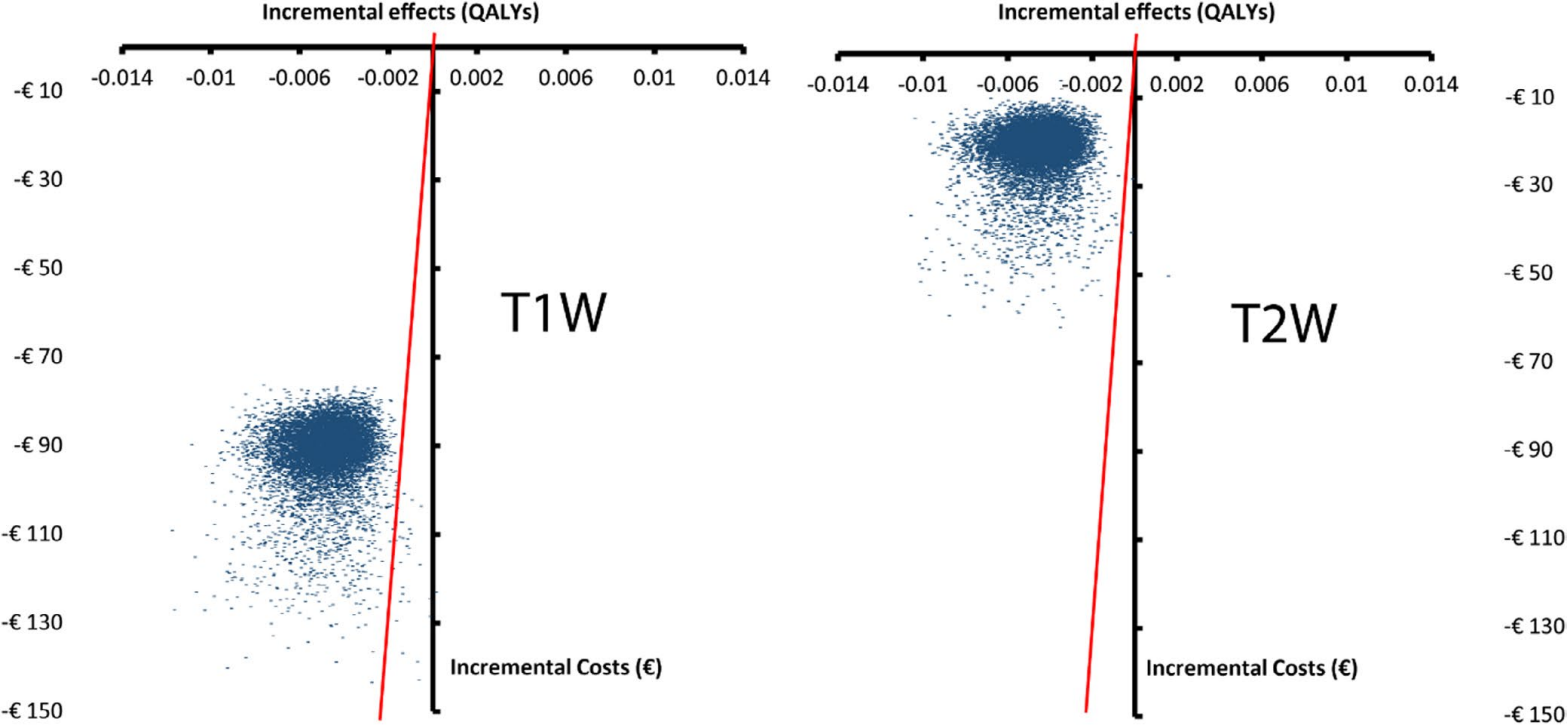
Scatter plot of probabilistic sensitivity analysis of the T1W scenarios (left) and the T2W scenario (right) displayed on the cost‐effectiveness plane. Every dot represents one of the 10.000 Monte Carlo simulations. The x‐axis describes the incremental effect in QALYs and the y‐axis the incremental cost of ABR‐MRI over standalone MRI in euros. In both scenarios, ABR‐MRI saved costs but was also less effective in most cases. The red line depicts a hypothetical willingness‐to‐accept threshold of €80 000 per QALY lost; the dots located right of the line were cost‐effective at this threshold

The scatterplot of the probabilistic sensitivity analysis showed that in nearly all simulations ABR‐MRI was not cost‐effective because of the limited cost‐saving and health loss (Figure 3). At a hypothetical threshold of €80 000 per QALY lost, standalone MRI was cost‐effective in 99.8% of the simulations in the T1W scenario and 99.9% in the T2W scenario.

## 4 DISCUSSION

Our study suggests that the limited cost‐saving with ABR‐MRI does not outweigh the number of missed patients with VS, and other important pathologies (resulting in QALY loss) that would have been detected when using standalone MRI. The analyses appeared to be sensitive to the probability that a missed patient was detected during follow‐up and the health‐related quality of life of a missed patient. The cost‐saving with ABR‐MRI was only worthwhile in scenarios with no negative consequences for missed patients.

Several other studies have performed cost (‐effectiveness) analyses on ABR‐MRI, but results were conflicting. Both Rafique et al. and Cueva found that ABR was not cost‐effective as a screening tool for VS due to the low sensitivity and specificity of ABR.^6^, ^10^ While Rupa et al. and Robinette et al. concluded that ABR is a cost‐effective strategy for preliminary screening of patients with VS‐like symptoms.^7^, ^12^ These studies, however, used small patient populations and had limited follow‐up.

In contrast to previous studies, our study is the first cost‐effectiveness analysis that compared ABR‐MRI with standalone MRI and included the long‐term effects of a missed case of VS. We made a solid comparison between ABR‐MRI and standalone MRI by dividing the population into three subgroups. Due to this method, the potential added value of MRI in detecting other pathologies than VS was included in our analyses. Furthermore, we distinguished different tumour sizes using the Koos classification to differentiate between the varying reported sensitivities and specificities of ABR.^5^, ^6^ This classification also allowed us to include the consequences of treatment of Koos 3 and 4 in our model.

Some limitations of our study should also be discussed. First, if we assumed a willingness‐to‐accept threshold of €80 000 per QALY lost, ABR‐MRI was not deemed cost‐effective. This threshold is derived from the willingness‐to‐pay threshold but currently, there is no consensus on the optimal method to determine the willingness‐to‐accept threshold. Nevertheless, the ICERs in this study generally fell below €25 000 per QALY lost indicating that the limited saving with ABR‐MRI does not outweigh lost QALY. Second, we assumed a small disutility (a lower HRQoL) for missed patients with OIP. The univariate sensitivity analysis showed that a disutility for these patients results in standalone MRI being cost‐effective. Only when missing a patient with OIP does not have a negative effect on the HRQoL, ABR‐MRI was potentially cost‐effective. Third, we assumed that patients received annual MRI screening after a VS diagnosis, but this might not be the most cost‐effective screening strategy. Instead, other monitoring frequencies might be more suitable. A previously published cost‐effective study showed that lifelong annual monitoring was most effective compared to five other monitoring strategies, but results were uncertain and it remains unclear if other monitoring frequencies might provide more value for money.^14^ However, varying the MRI screening frequency (biennial or quinquennial) did not alter our conclusions.

According to our study, standalone MRI is cost‐effective mainly because other pathology is missed when using the ABR‐MRI strategy. Using standalone MRI as the primary detection method increases medical costs, but we argue that indirect costs arising from missing other pathology and further diagnostics are potentially being prevented. Limited healthcare resources might be better spent on methods that can detect a wide variety of diseases (like MRI), instead of diagnostic methods that limit to an unspecified set of diseases (like ABR). Given that ABR‐MRI saves costs compared to standalone MRI, the question remains if we are willing to drop the gold standard to save costs, and how we determine the level of effect that we are willing to sacrifice at what price. There might still be a role for ABR in the detection of VS because not all patients want or can undergo MRI scans and thus ABR might provide an alternative option in shared decision making.

In conclusion, the cost‐saving with ABR prior to MRI does not seem to outweigh the number of missed patients with VS and other important pathologies that would have been detected when using standalone MRI for the diagnosis of patients suspected of a vestibular schwannoma.

## CONFLICTS OF INTEREST

None to declare.

## AUTHOR CONTRIBUTION

MH, HK and MR designed the study; SW built the model; MH, AB and HK provided model input; SW and MH drafted the manuscript; SW, MH, AB, HK and MR revised and approved the manuscript; SW, MH, AB, HK and MR agree to be accountable for all aspects of the work.

## Supporting information

Appendix S1

## Data Availability

The data that support the findings of this study are available from the corresponding author upon reasonable request.
