# Supplementary material for: Auditory brainstem response prior to MRI compared to standalone MRI in the detection of vestibular schwannoma: A modelling study
Source: Clin Otolaryngol. 2021 Nov 24;47(2):295–303. doi: 10.1111/coa.13894 (PMC9298692; doi:10.1111/coa.13894)
Supplement: Supplementary file 1 — Appendix S1 [file COA-47-295-s001.docx]

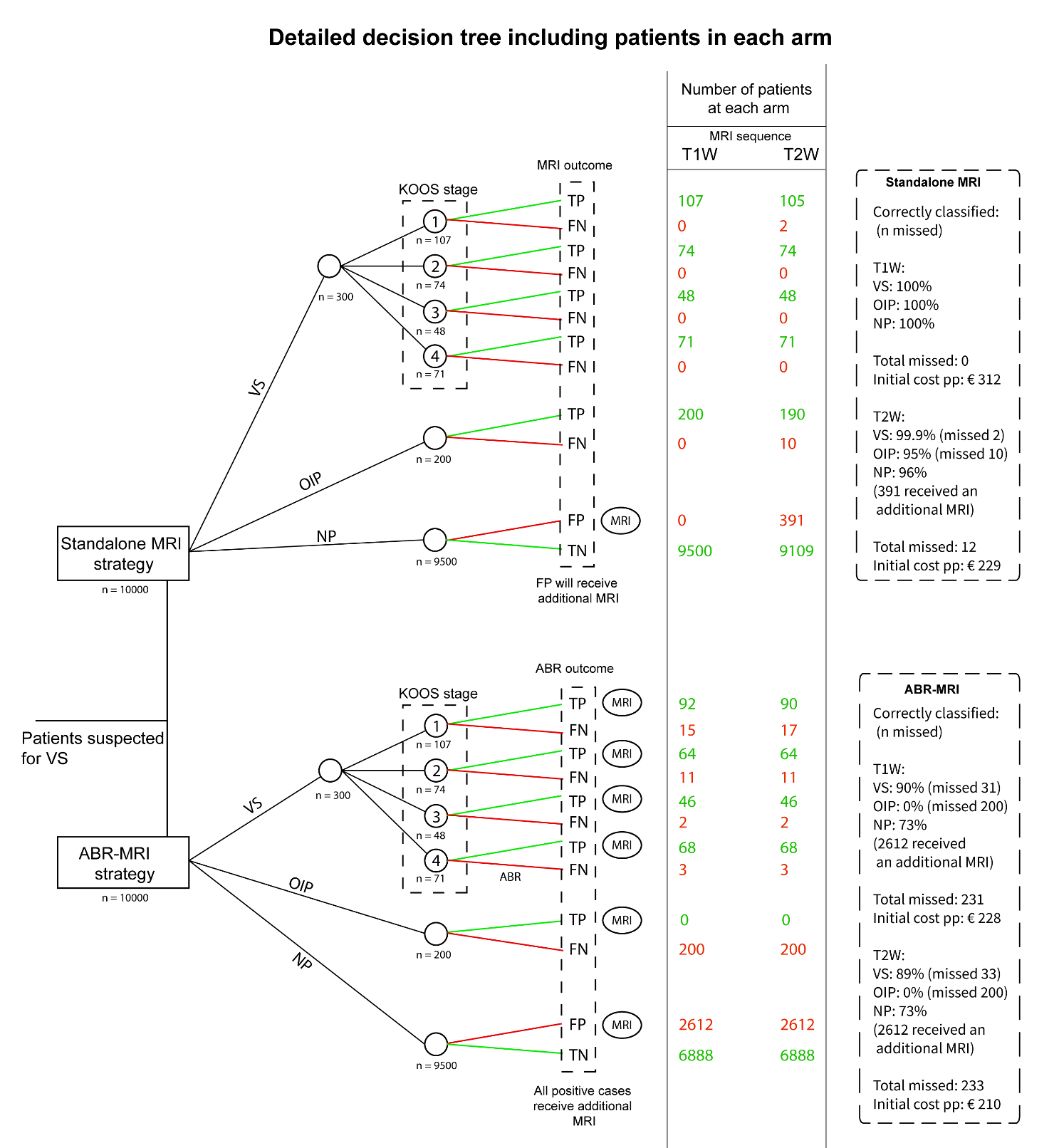
**APPENDIX I: Detailed decision tree including patients in each arm**

A cohort of 10.000 patients was simulated for both strategies (Standalone MRI & ABR-MRI) and categorized based on the underlying disease (either a vestibular schwannoma (VS), other important pathology (OIP) or no pathology (NP)). Patients with VS were split based on KOOS stage (tumor size). Subsequently, patients receive either MRI or ABR. Patients with VS or OIP can be detected (true positive; TP) or missed (false negative; FN). Patients with NP can be classified as truly negative (TN) or as false positive (FP). All patients with a positive ABR (TP and FP) received an additional MRI.
